# Management of tunica albuginea disruption secondary to malleable penile implant fracture: insights into etiology and surgical approach

**DOI:** 10.1186/s12610-025-00276-z

**Published:** 2025-08-01

**Authors:** Emad S Rajih

**Affiliations:** 1 Urology Division, General and Specialized Surgery Department, College of Medicine, Taibah University, Madinah, Saudi Arabia; 2 Urology Section, Department of Surgery, King Faisal Specialist Hospital and Research Center, Madinah, Saudi Arabia

**Keywords:** Erectile dysfunction, Treatment, Corporoplasty, Tunica Albuginea, Dysfonction erectile, Traitement, Corporoplastie, Tunique albuginée

## Abstract

**Background:**

The tunica albuginea is a key anatomical structure supporting penile implants. Several factors can lead to anatomical penile defects, contributing to penile prosthesis malfunction.The aim is to describe the consequences of malleable penile implant fractures, their contribution to tunica albuginea defects, and management outcomes.

**Results:**

All patients underwent malleable device surgery were reviwed retrospectively in our center. We included three patients with malleable penile implant disruption. Two patients underwent concomitant plication corporoplasty (PC) with device replacemen and one patient with device replacement only that showed intact tunica albuginea. The mean age at revision was 71.3 years. All patients achieved device stability and reported successful vaginal penetartion after PC and device replacement, with a median follow-up of 28 months.

**Conclusions:**

Malleable penile device fractures are extremely rare findings. They can cause tunica albuginea disruption and tears, compromising penile penetration and device stability. Device replacement combined with PC is an essential component of successful vaginal pentration.

## Résumé

**Contexte:**

La tunique albuginée est une structure anatomique clé qui soutient les implants péniens. Plusieurs facteurs peuvent entraîner des anomalies anatomiques du pénis, contribuant à une dysfonction de la prothèse pénienne. L’objectif est de décrire les conséquences des fractures de l’implant pénien malléable, leur contribution aux anomalies de la tunique albuginée et les résultats de la prise en charge.

**Résultats:**

Tous les patients ayant subi une chirurgie pour dispositif malléable ont été examinés rétrospectivement dans notre centre. Nous avons inclus trois patients présentant une rupture de l’implant pénien malléable. Deux patients ont subi une corporoplastie de plicature concomitante avec remplacement du dispositif, et un patient avec remplacement du dispositif uniquement en raison de la présence d’une tunique albuginée intacte. L’âge moyen au moment de la révision était de 71,3 ans. Tous les patients ont atteint une stabilité du dispositif et ont signalé une pénétration vaginale réussie après la mise en place d’une corporoplastie de plicature et du dispositif, avec une médiane de suivi de 28 mois.

**Conclusions:**

Les fractures de dispositif malléable pénien sont des découvertes extrêmement rares. Elles peuvent provoquer des perturbations et des déchirures de la tunique albuginée, compromettant la pénétration pénienne et la stabilité du dispositif. Le remplacement du dispositif combiné à une corporoplastie de plicature est un élément essentiel d’une pénétration vaginale réussie.

## Background

Male erectile dysfunction (ED) is a prevalent condition, affecting 52% of men aged 40 to 70 years according to the Massachusetts Male Aging Study [1]. Penile implants are considered the standard of care for patients with ED refractory to medical therapy [2]. During the past four decades, advancements in the synthetic properties of penile implants have aimed to enhance device durability and function. These improvements have reduced the incidence of penile structural deformities resulting from prosthesis damage [2–4].

Penile structural abnormalities, particularly in the tunica albuginea, have been evaluated in various studies. These evaluations suggest that such penile structural abnormalities may affect device function, integrity, and stability [3–5]. However, these penile abnormalities remain incompletely understood. Various reports have described penile structural defects secondary to penile implant surgery, including urethral erosion, penile wobble effect due to proximal corporal deformity, and/or corpora cavernosa erosion [4–6]. Moreover, some reports suggest that the intrinsic histological features of the tunica albuginea may influence these findings [7, 8]. Notably, most penile structural abnormalities described in the literature are associated with inflatable penile prostheses [4]. Isolated proximal penile deformity could result from affected inflatable penile prosthesis with aneurysmal defect that need corrective surgical repair with device replacement [5]. In this report, we describe the occurrence of device instability secondary to malleable penile implant damage in a series of three patients, necessitating device replacement and corporal reconstruction.

## Materials and methods

### Patient selection

Following institutional review board approval with approval number TU-25-031, a retrospective review was conducted using a penile implant database encompassing surgeries performed between May 2019 and March 2024. All patients underwent penile device implantation for erectile dysfunction were recorded in the penile implant database. The procedures were performed by a single surgeon (ESR) at three medical centers (two public and one private facility). Patient information and data were documented in the institutional electronic medical records at time of surgery and follow-up visits. Retrospectively, patient characteristics and study variables were extracted from the medical records in a standardized database, with a maximum follow-up period of 6 months. Approximately 23% of patients underwent follow-up for >1 year.

All cases of malleable penile device revisions were recorded during penile prosthesis revision surgeries. Based on the clinical presentation, malleable penile device fractures and tunica albuginea disruptions were assessed perioperatively by identifying device instability and penile angulation with a hinge effect that preclude vaginal pentration, confirmed intraoperatively by the presence of tunica albuginea disruption. Cases involving tunica albuginea reconstruction for indications other than malleable penile device fractures were excluded.

### Covariates

Patient characteristics and variables were recorded, including age, comorbidities, erectile dysfunction etiology (based on clinical assessment), the number and duration of prior penile implants, and the duration of previous implant failure. Intraoperative findings included the types of explanted and implanted devices, affected cylinders and laterality, previous and current implant measurements, and affected corpora. Postoperative outcomes, including successful sexual intercourse with vaginal penetration and follow-up duration, were also documented.

### Surgical management

Malleable penile device replacement with concomitant plication corporoplasty was performed during revision surgery. Following initial clinical assessment and confirmation of device fracture, a penoscrotal approach was used for all revisional surgery. The damaged penile prothesis and their rear tip extenders were removed. The explanted device examined for any abnormal defects. The site of the device fracture was identified in the current series. Additionally, the site of tunica albuginea defects was also identified that were corresponding to site of damaged penile implant at the mid part. The penoscrotal incision was sufficient to access the tunica defect. The neurovascular bundles were mobilized at the affected side prior to plication corporoplasty. Then, interrupted 3-0 polydioxanone sutures (Ethicon, Inc., Bridgewater, NJ, USA) were applied to plicate the tunica albuginea at the affected site. Antimicrobial irrigation was performed before placing the new device. The product of the newly implanted device is based upon the availability in the center. Corporotomies were closed with a continuous 2-0 Vicryl suture (Ethicon, Inc.). Patients were advised to resume sexual intercourse 2 months postoperatively.

## Results

**Fig. 1:**
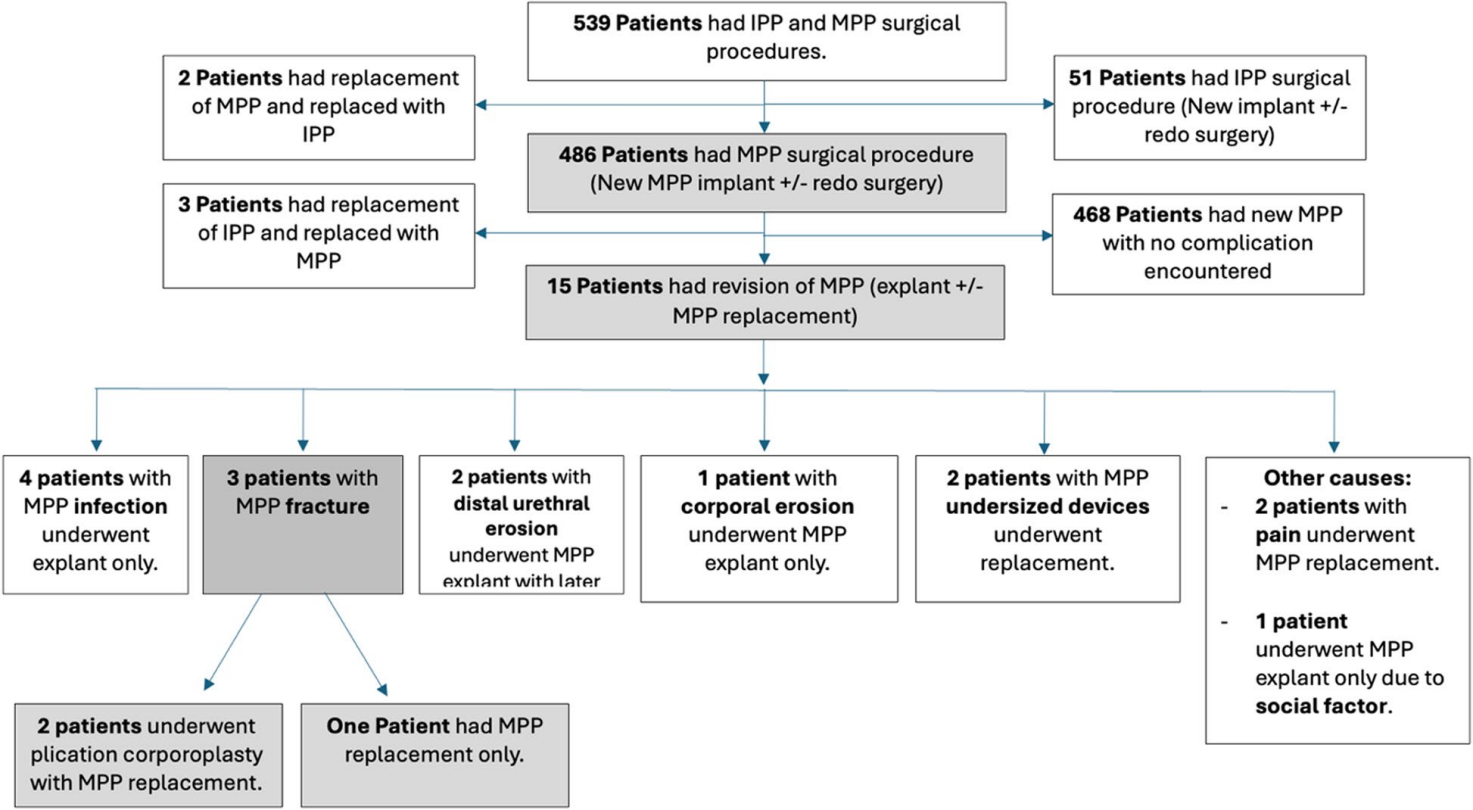
Flowchart demonstrating selection of patients from the penile implant database. IPP, inflatable penile prosthesis; MPP, malleable penile prosthesis

**Fig. 2:**
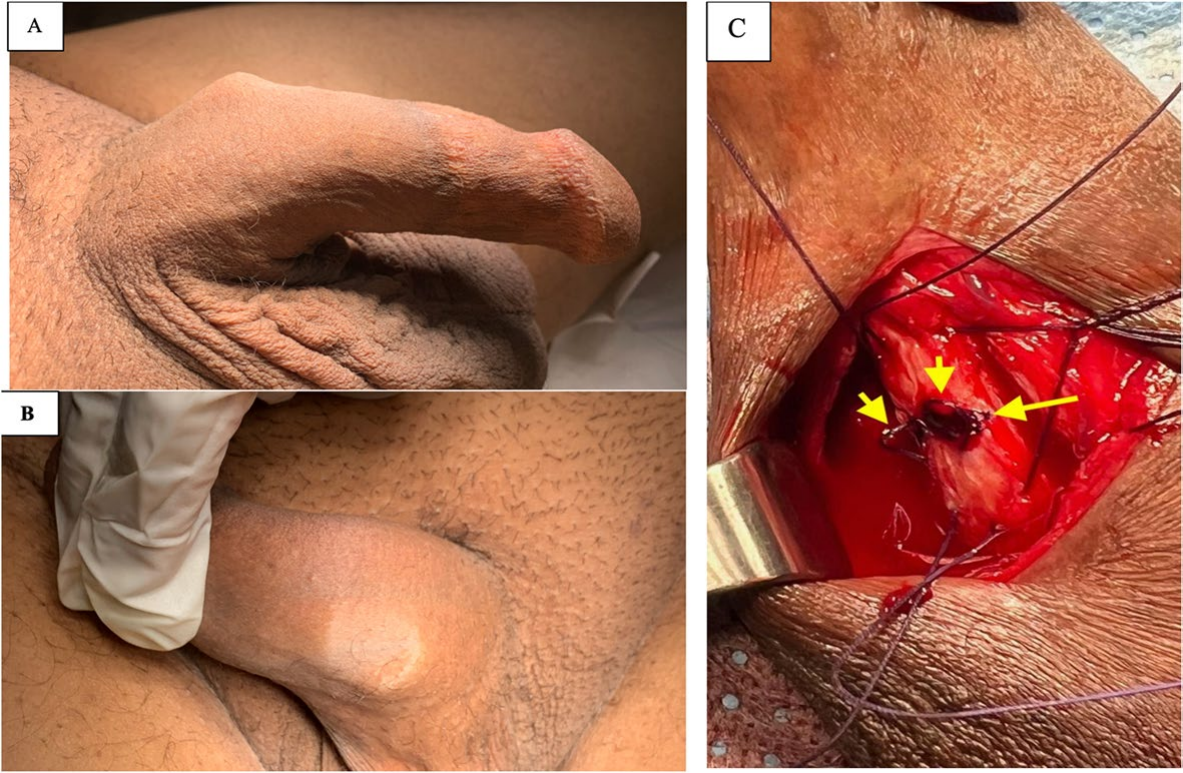
Photographs of Patient 1. **A**, **B** Preoperative photographs showing the angulation site of the affected penis, with penile angulation and device erosion through the left tunica albuginea beneath the skin. **C** Intraoperative image showing plication sutures (short arrows) applied to the weakened tunica wall (long arrow)

In the penile implant database, we identified 15 patients who underwent malleable penile device replacement. Of these, three patients presented with a fractured penile device with or without tunica albuginea disruption with a hinge effect, necessitating plication corporoplasty and device replacement (Figure 1). All three patients with fractured devices reported difficulty with penile penetration at the time of initial presentation, which was secondary to penile angulation during penetration (Figure 2). The baseline patient characteristics are shown in Table 1. The median age at the time of surgical management for patients with malleable penile fractures was 64 years. The etiology of erectile dysfunction was consistent across all patients, with arterial insufficiency secondary to diabetes, hypertension, and ischemic heart disease.

**Table 1:** Demographic and clinical characteristics

Patient	Age (years)	Comorbidities	Etiology of ED	Past implant	Implant failure time	Duration of previous penile implant
1	59	DM, DSL	Arterial insufficiency	Once	15 months	6 years
2	81	DM, HTN, IHD	Arterial insufficiency	Twice	24 months	14 years
3	74	DM, HTN, IHD	Arterial insufficiency	Once	6 months	7 years

*ED* erectile dysfunction, *DM* diabetes mellitus, *DSL* dyslipidemia, *HTN* hypertension, *IHD* ischemic heart disease

**Fig. 3:**
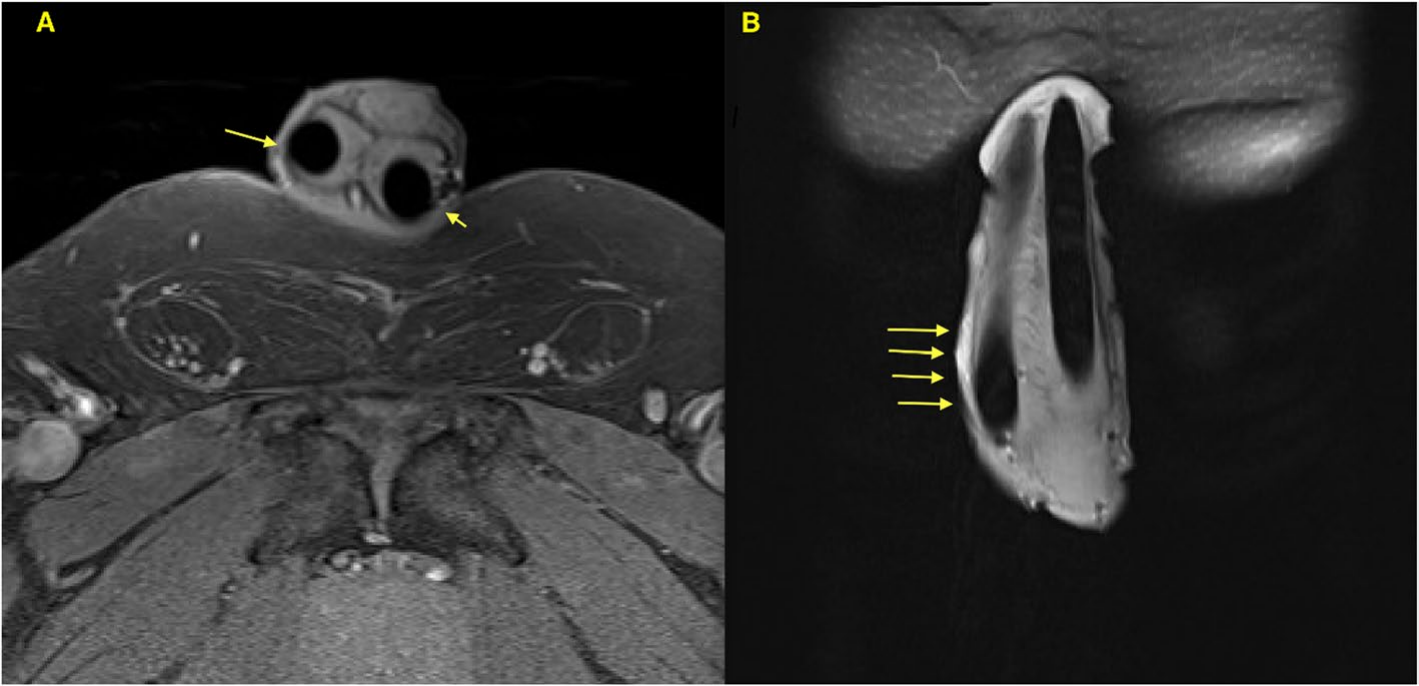
Penile magnetic resonance images of Patient 2 following initial device replacement without plication corporoplasty. **A** Axial view showing thin and weakened tunica albuginea bilaterally (arrows). **B** Coronal view highlighting the device angulation site at the right corpora cavernosa (arrows)

Detailed clinical and intraoperative findings revealed that one patient (Patient 2) had undergone a prior penile implant revision that was unsuccessful and required further revision with corporoplasty. Additionally, a magnetic resonance imaging study performed before the final revision showed bilateral thinning of the corporal wall (Figure 3). The last implanted device was intact; however, plication corporoplasty had not been performed.

**Fig. 4:**
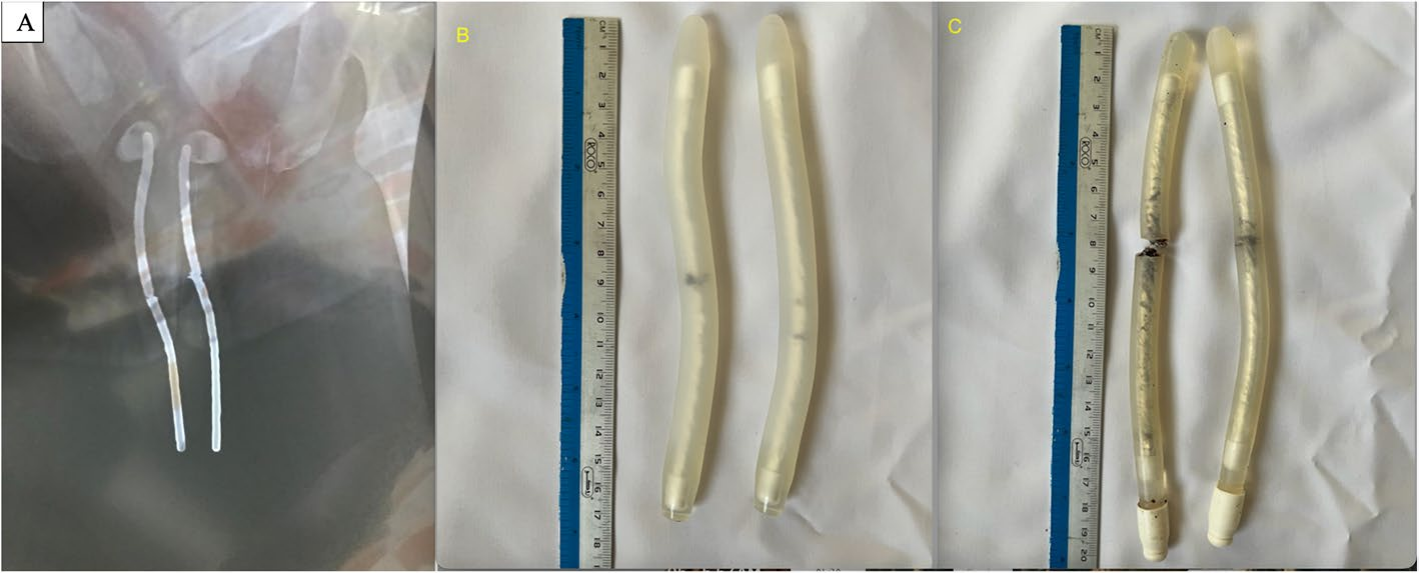
Radiographic and intraoperative findings of fractured malleable penile implants. **A** Penile anteroposterior X-ray of Patient 1 showing bilateral silver wire fractures. **B** Explanted penile device from Patient 3, demonstrating separation of silver wires with intact silicone covering. **C** Explanted penile device from Patient 1, showing separation of both silver wires and the silicone tube on one side

The fractures involved both implanted cylinders in all patients at the same level, affecting the metallic core at the site of the penile base. In two of the six cylinders, the metallic core had eroded through the outer covering material (Figure 4). Detailed operative findings are presented in Table 2.

**Table 2:** Operative findings

Patient	Explanted damaged MPP (girth)	Silver wire disruption side	Silicone tube disruption side	Previous implant measurements	Current implant measurement	Affected corpora	Reimplanted MPP (girth)	Follow-up
1	Promedon (11 mm)	Bilateral	Left side	20 cm	22 cm	Left side	Tactra™ (13 mm)	30 months
2	Promedon (11 mm)	Bilateral	Bilateral	19 cm	19.5 cm	Bilateral	Genesis^®^ (13 mm)	26 months
3	Promedon (13 mm)	Bilateral	Intact	17 cm	18 cm	Intact	Zephyr ZSI 100 (13 mm)	6 months
4								

*MPP* malleable penile prosthesis

The median follow-up time after device replacement and corporoplasty was 26 months. All patients reported satisfactory sexual intercourse with no complications or need for additional surgery.

## Discussion

In this report, we present a case series of three patients who developed dysfunction of malleable penile prostheses secondary to fractured implants, involving both the central silver wire and the covering silicone tube. These fractures compromised the structural integrity of the penis, as evidenced during clinical evaluation through patient history and physical examination, which revealed an angled penile implant and weakening of the tunica albuginea at the fracture site. Imaging studies further aided in identifying the etiological factors contributing to the device dysfunction. Surgical management included device replacement and plication corporoplasty at the defect site. During follow-up, the malleable devices remained functional, with no recurrence of the hinge effect.

This abnormality was observed in patients with gradually progressive dysfunctional penile penetration due to long-standing fractured malleable penile implants, rather than sudden acute fractures occurring during sexual intercourse. Recognizing this condition requires an understanding of its characteristics to ensure timely and appropriate management. It may be justifiable to perform additional imaging workups, such as conventional X-rays and magnetic resonance imaging, as adjuncts before surgical revision to evaluate the need for device replacement and possible plication corporoplasty.

In this series, three patients experienced dysfunctional sexual intercourse, requiring hand assistance for penile penetration. All patients had undergone prior evaluations, during which the etiological factor for their condition was not initially recognized. We postulate that early detection of concomitant corporal defects, combined with timely device replacement and plication corporoplasty, might have prevented the surgical failures observed in one patient in this series.

Late presentation with sexual dysfunction following penile implant surgery has been reported in the literature, with various etiological factors attributed to device abnormalities and/or penile anatomical issues [5, 9]. Abnormalities of inflatable devices include aneurysmal dilation, cylinder breakage, fluid leakage, and pump failure. Notably, mechanical failure of malleable devices due to device breakage is extremely rare and has been documented only in case reports [10–15]. In the current study, we report the first documented observation of concomitant malleable device fractures with tunica disruption in two of three patients, necessitating plication corporoplasty.

Tunica albuginea defects in penile implant surgery have been reported in the literature in association with various anatomical abnormalities. These penile abnormalities may occur primarily due to proximal corporal dilation or secondarily as a result of inflatable penile device abnormalities, which contribute to corporal cavity dilation and wobble penile defects [5, 7, 16]. In the current study, tunica albuginea defects occurred secondary to device angulation at the site of malleable device fractures, which eroded into the corpora cavernosa wall were it is a vulnerable area as described by Osmonov et al in cadaveric study [17].

Recent literature showed limited number of corporal deformity secondary to penile prothesis failure and damage. These include capsular contaction, proximal corporal dilatation, and peniel wobble deffect. Those patients were treated with corporoplasty and device replacement. And all reported cases showed greater penile stability and satisfactory function. These findings validate the improvement of sexual function when secondary structural penile deformity discovered and managed appropriatly.

Interestingly, all the revisional cases involving tunica disruption and device fracture in our series were associated with the Tube®-Promedone (Promedon, Córdoba, Argentina) device [18]. This device is characterized by a central silver twisted wire core, which ensures axial rigidity and malleability. The central silver core is covered by a PTFE (polytetrafluoroethylene) layer to secure the implant. Additionally, the outer layer of the silver-PTFE structure is encased in a tapered, biocompatible silicone elastomer.

The flexible silicone elastomer features a soft distal segment, a medium-hardness middle segment, and a high-hardness proximal segment surrounding the silver core wire. This design provides the malleable device with unique flexibility, enabling the patient to bend the implant up to 130 degrees [18]. However, this property may pose a long-term risk to the integrity of the device, and further research is required to establish a definitive association.

Mohamed et al. conducted a retrospective study involving 128 patients who underwent implantation of the TUBE malleable device, with both short- and long-term follow-up. They reported three cases (2.3%) of mechanical failure due to wire fracture occurring at 13, 19, and 22 months post-surgery [19]. In our current study, we report device failure over a longer term, exceeding five years, as detailed in Table 1. Furthermore, Mohamed et al. [19] did not report any concomitant tunica tears. This may be attributed to the intact PTFE covering and the relatively short interval between device implantation and wire fracture. The application of plication corporoplasty is effective and safe option for treating penile deformity as showed in our series when present secondary to device abnormality as described in previous studies [20].

### Study limitations

This report has certain limitations, including the small number of patients in this case series. However, given the high volume of malleable penile prosthesis insertions in the region, the rarity and unusual nature of these findings are noteworthy. Despite this limitation, future studies could further investigate the predisposing factors for tunica albuginea disruption, including histological analysis and evaluation of device manufacturing properties. In the current case series, we did not examine the histological detail of the tunica albuginea with collagen and elastin fibers distribution to examine the association. Additionally, the use of adjunct tunica albuginea-supporting grafts alongside corporoplasty may improve long-term outcomes following repair and help prevent future device fractures and tunica weakness at the site of corporoplasty repair. However, this approach requires long-term follow-up to validate its efficacy. The current report highlights the importance of recognizing this phenomenon to enable andrologists to identify pathological tunica albuginea weakness, facilitate immediate repair during device replacement, and avoid incomplete surgical management, thereby improving the likelihood of achieving satisfactory sexual intercourse.

## Conclusion

The integrity of the tunica albuginea is crucial for achieving optimal sexual function with a malleable penile device. Fracture of a malleable penile device can lead to tunica albuginea disruption, compromising satisfactory sexual intercourse and vaginal penetration. Device replacement combined with or without corporoplasty is an essential component of the successful surgical management for this phenomenon.

## Data Availability

No datasets were generated or analysed during the current study.
